# Supplementary material for: All-optical scattering control in an all-dielectric quasi-perfect absorbing Huygens’ metasurface
Source: Nanophotonics. 2022 Dec 15;12(1):139–46. doi: 10.1515/nanoph-2022-0597 (PMC11501191; doi:10.1515/nanoph-2022-0597)
Supplement: Supplementary file 1 — Supplementary Material Details [file j_nanoph-2022-0597_suppl.pdf]

Supplementary Materials:

All-optical scattering control in an all-dielectric quasi-perfect absorbing Huygens' metasurface

Kentaro Nishida[1], Koki Sasai[2], Rongyang Xu[2], Te-Hsin Yen[1], Yu-Lung Tang[1], Junichi Takahara[2,3,], and Shi-Wei Chu[1,4,5,*]*

[1] Department of Physics, National Taiwan University, No. 1, Sec. 4, Roosevelt Rd., Taipei 10617, Taiwan

[2] Graduate School of Engineering, Osaka University, 2-1 Yamadaoka, Suita, Osaka 565-0871, Japan

[3] Photonics Center, Graduate School of Engineering, Osaka University, 2-1 Yamadaoka, Suita, Osaka 565-0871, Japan

[4] Molecular Imaging Center, National Taiwan University, No. 1, Sec. 4, Roosevelt Rd., Taipei 10617, Taiwan

[5] Brain Research Center, National Tsing Hua University, 101, Sec 2, Guangfu Road, Hsinchu 30013, Taiwan

(*Corresponding authors)

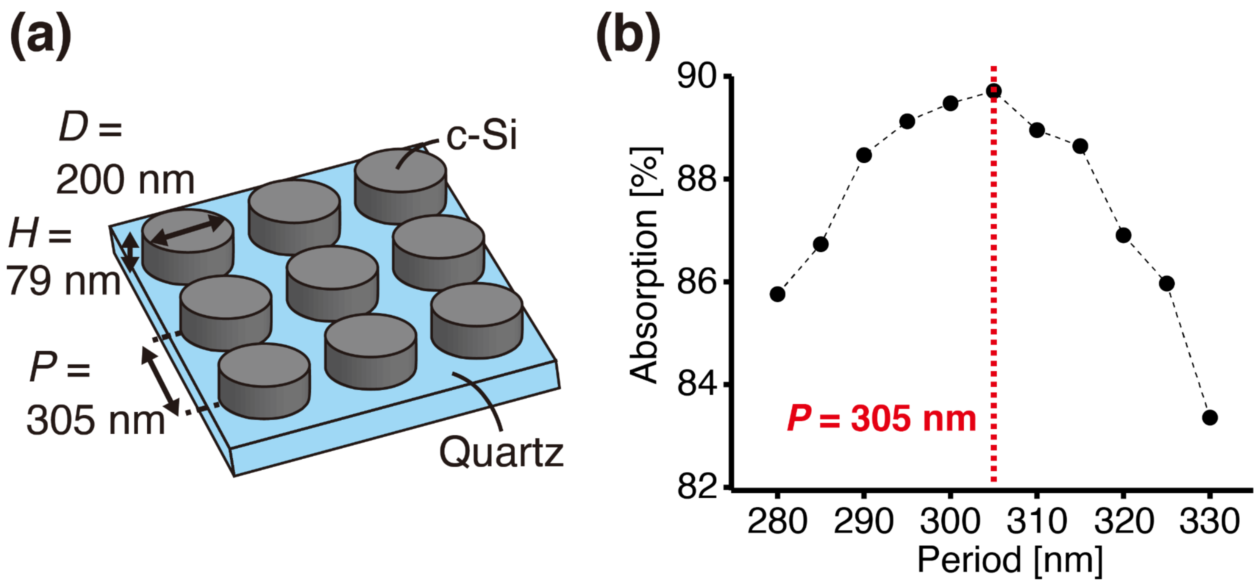

Figure S1: (a) Structure of our silicon metasurface. (Abbreviation, D: diameter, H: height, P: period, c-Si: crystalline-silicon) (b) Relationship between the period of silicon nanodisc meta-atoms and absorption. The diameter and the height of silicon nanodisc meta-atoms are 200 nm and 79 nm, respectively. The illumination wavelength is 561 nm. The absorption is maximized at the period of 305 nm, which is same size as our silicon metasurface.

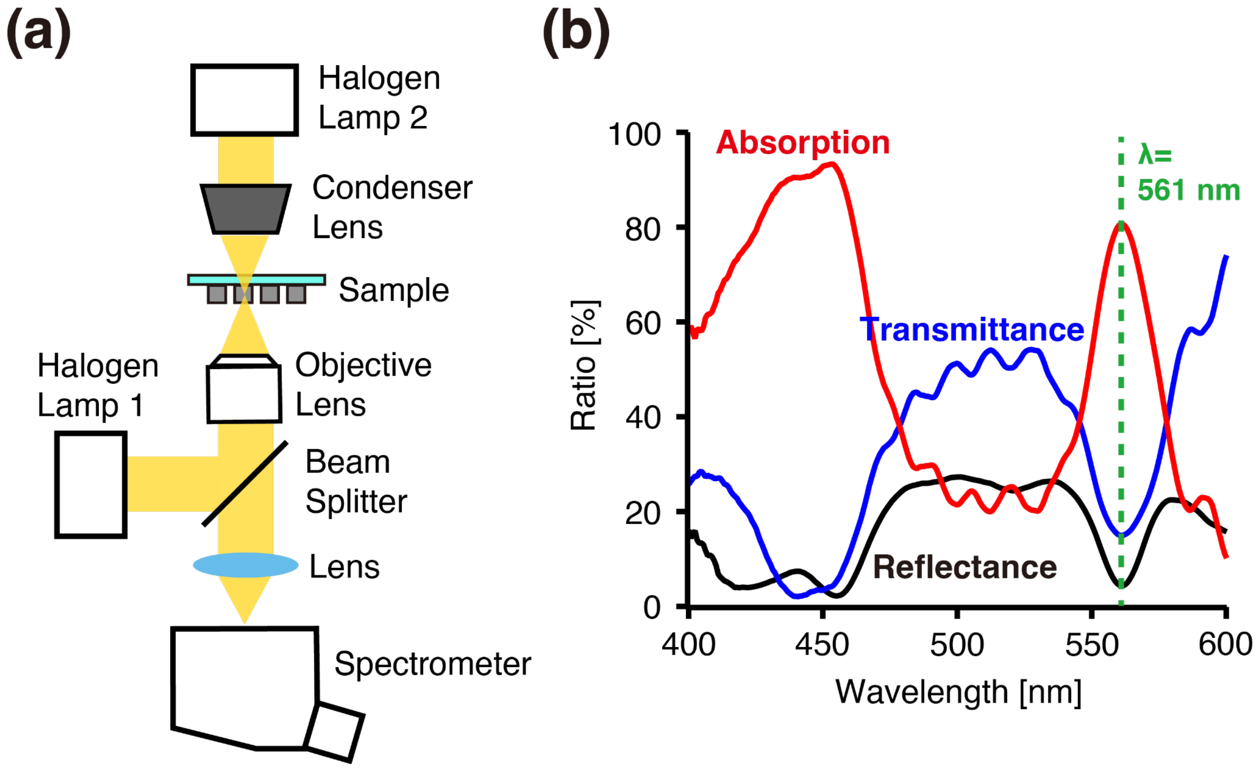

Figure S2: (a) Optical setup for spectral measurement. The reflectance and transmittance spectra from the sample were measured by using illumination from backward (Halogen lamp 1) and forward (Halogen lamp 2) directions, respectively. (b) Spectral properties of the silicon metasurface. Black, blue and red solid lines indicate reflectance, transmittance and absorption spectra, respectively. The diameter, period, and height of the silicon nanodiscs meta-atoms in the metasurface are 200 nm, 305 nm, and 79 nm, respectively. The absorption spectrum was calculated by 100%-T-R, where T and R respectively indicate transmittance [%] and reflectance [%]. Our silicon metasurface exhibited absorption of 80.6 % and transmittance of 15.0 % at the wavelength of 561 nm. Note that the absorption by the substrate is estimated to be negligibly small (~10^{-3} %).

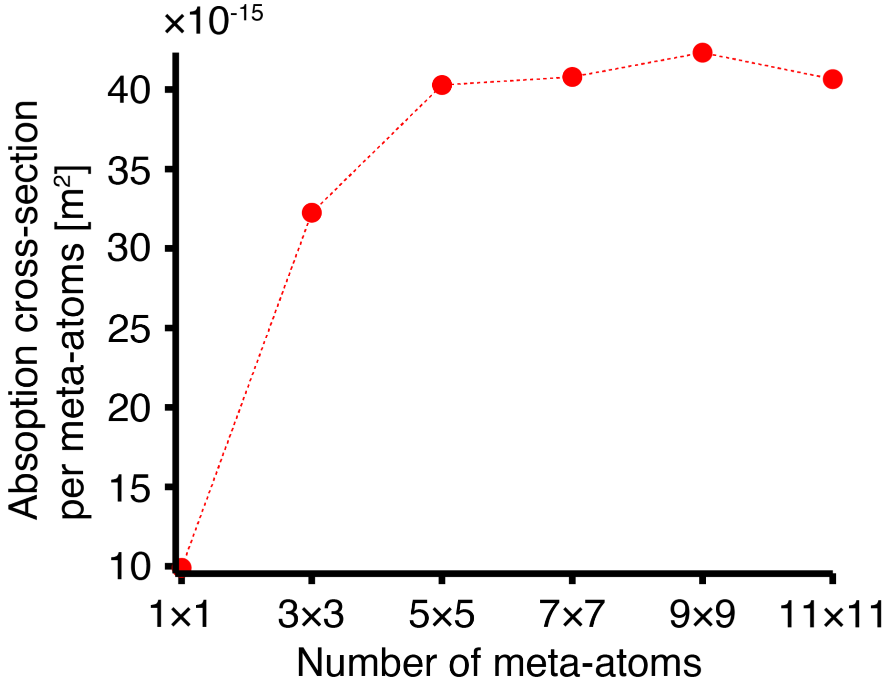

Figure S3: Relationship between absorption cross-section of meta-atoms and the number of illuminated silicon nanodisc meta-atoms. The diameter, period, and height of silicon nanodiscs in the metasurface are 200 nm, 305 nm, and 79 nm, respectively. Excitation wavelength is 561 nm. The absorption cross-section of each meta-atoms significantly decreases, when the number of silicon meta-atoms illuminated by the incident beam is lower than 5×5.

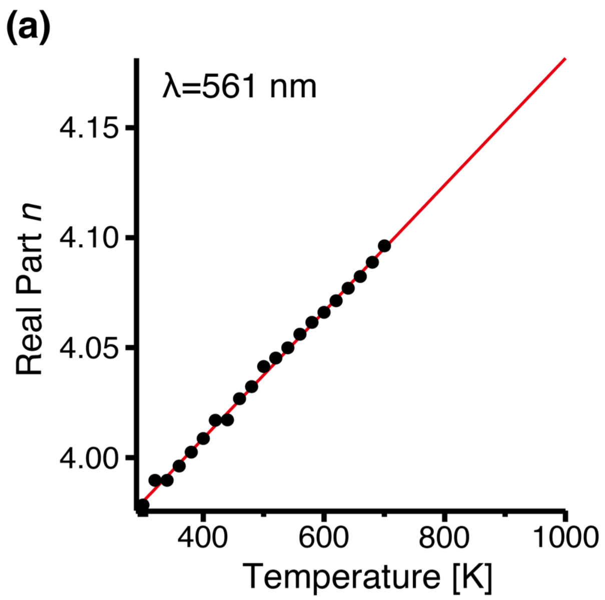

Figure S4: Dependences of real (a) and imaginary (b) refractive indexes of crystalline silicon at the wavelength of 561 nm. The black dots indicated experimentally measured data by using a commercial ellipsometer equipped with a sample heater (M-2000 DI ellipsometer, JA Woollam). The red solid line indicates the extrapolation curve.

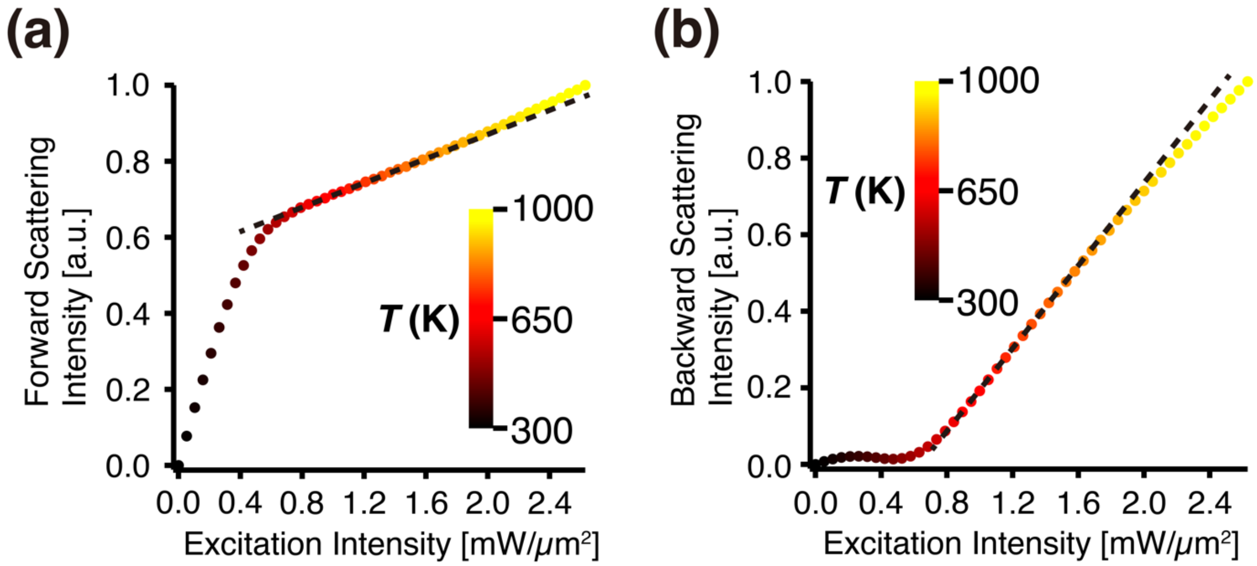

Figure S5: Theoretically calculated dependences of (a) forward and (b) backward scattering intensities from silicon metasurface on the excitation power, for a high excitation power region. Excitation wavelength of 561 nm. The diameter, period, and height of silicon nanodiscs meta-atoms in the metasurface are 200 nm, 305 nm, and 79 nm, respectively. The number of illuminated meta-atoms is 9×9. The color of the plot indicates the equilibrium temperature (K) under light irradiation. Dotted lines indicate proportional increase to excitation intensity. The forward and backward scattering intensity respectively show gradual reverse-saturation and saturation effect, when the excitation intensity is increased up to ~2.0 mW/μm^2 and the temperature reaches ~920 K,

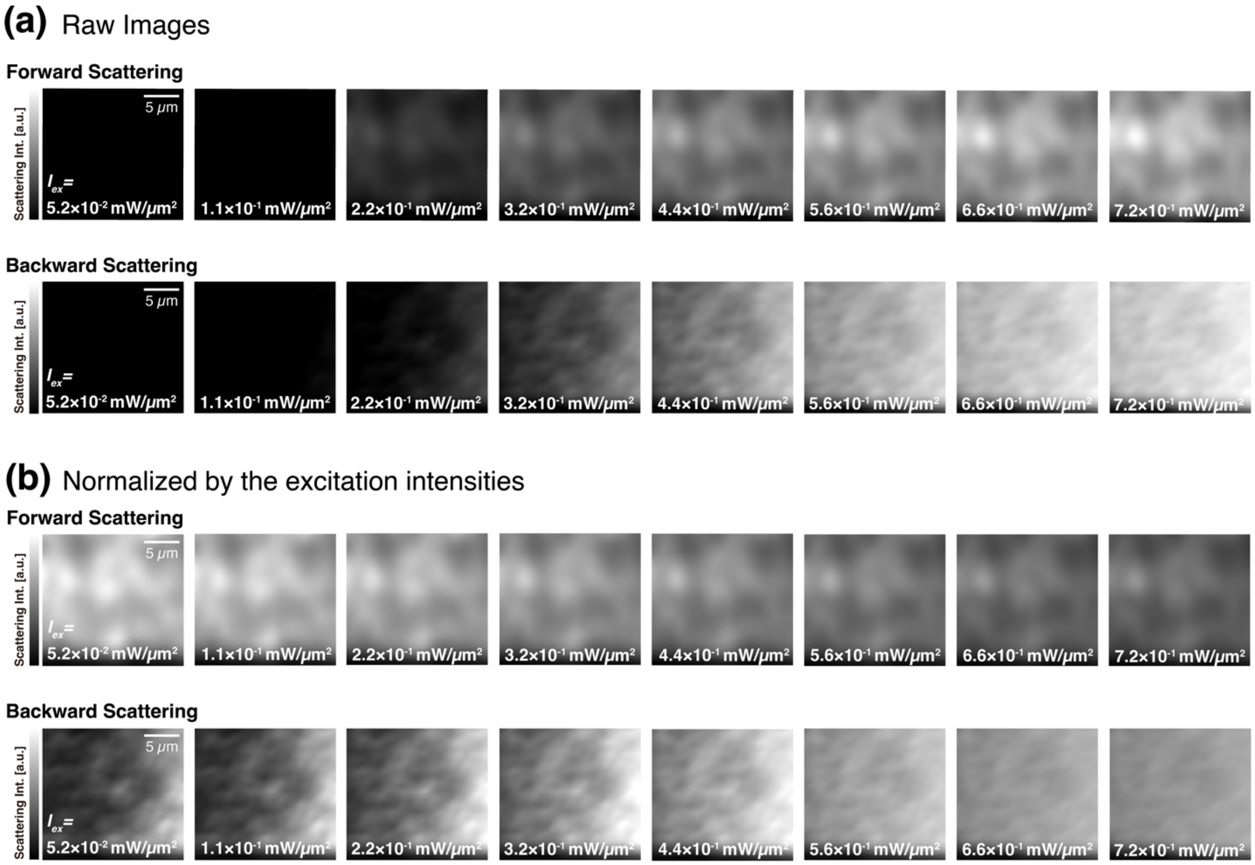

Figure S6: (a) Forward and backward scatterings images of silicon metasurface at various excitation intensities. The excitation wavelength is 561 nm. The NA 0.4 dry objective lens was used for illumination. The pixel size and dwell time were respectively 180 nm and 12 µs. (b) Normalized images by the excitation intensities. The normalized forward scattering images are getting darker as the excitation intensity increases, indicating the sub-linear trend of scattering response. On the other hand, in the case of normalized backward scattering images, the images get brighter due to the super-linear increase of the scattering intensity. After the excitation intensity reaches above 5.6×10^{-1} mW/µm^2, the brightness of the images does not increase anymore, because of the saturation effect.
